# First report of neonatal-onset glutaric aciduria type II in the Iranian population caused by a novel deleterious *ETFA* variant

**DOI:** 10.1186/s13023-025-04107-2

**Published:** 2025-11-18

**Authors:** Farshid Parvini, Mobarakeh Ajam-Hosseini, Marziyeh Shadpour

**Affiliations:** 1 Department of Biology, Faculty of Basic Sciences, Semnan University, Semnan, Iran; 2 Department of Molecular Genetics, Faculty of Biological Sciences, Tarbiat Modares University, Tehran, Iran

**Keywords:** *ETFA* gene, Glutaric acidemia type II, Neonatal-onset, Hypoglycemia, Acidosis, Iran

## Abstract

**Background:**

Glutaric acidemia type II (GA2), also known as multiple acyl-CoA dehydrogenase deficiency (MADD), is a rare inherited error of amino acid and fatty acid metabolism. Its clinical manifestations can vary from severe events that threaten the life of a newborn to milder and late manifestations. Here, we examined an Iranian couple for pre-pregnancy counseling who had a history of the death of two children suspected of metabolic disorder.

**Methods:**

Whole exome sequencing (WES) was performed to determine possible pathogenic genes in the parents of two deceased neonates. Sanger sequencing was then used to confirm the variant found. Subsequently, the possible impact of the identified variant on the ETFA protein was evaluated using bioinformatics tools.

**Results:**

WES identified a novel heterozygous in-frame variant c.485_493del: p.E162_T164del in exon 6 of the *ETFA* gene, which co-segregated with the autosomal recessive GA2 disorder in the family studied. Sanger sequencing confirmed the variant found in the parents and their healthy family members and in silico approaches showed disease-causing nature of the identified mutation. Following the confirmation of the identified variant in the fetus, a legal abortion permit was issued, and the fetus was terminated with the parents’ consent.

**Conclusions:**

This is the first reported case of GA2 caused by a variant in the *ETFA* gene in Iran and shows the importance of genetic diagnosis and management of rare clinical manifestations and conditions that can help predict prognosis and provide more accurate diagnostic information for patients and families with GA2.

## Background

Glutaric acidemia type II (GA2) or acyl-CoA dehydrogenase deficiency (MADD) is a rare congenital metabolic disorder with autosomal recessive inheritance caused by a defect in mitochondrial beta-oxidation and inefficient metabolism of amino acids and choline [1]. The GA2 frequency is estimated to be about 1/200,000 live births [2]. Electron transport flavoprotein (ETF) is a dimeric mitochondrial matrix protein composed of two subunits, alpha (ETFA) and beta (ETFB). ETFA protein has two domains and a FAD cofactor, which is essential for maintaining mitochondrial flavin balance [3]. GA2 is a heterogeneous disease. Several genes have been implicated in GA2 disease, including *ETFA*, *ETFB*, and ETF dehydrogenase (*ETFDH*) [4], which were mapped to 15p23–25, 19q13.1 and 4q33, respectively [5]. In 1977, Ruzicka et al., first demonstrated that ETF-QO acts as an electron acceptor for ETF and ubiquinone reductase [6]. ETF is an electron acceptor for dehydrogenases, and along with ETF-QO provide a short path to transfer electrons from several different mitochondrial adenine dinucleotides containing mitochondrial acyl-CoA dehydrogenases to ubiquinone [7]. Ubiquinone is located in the inner membrane of mitochondria and plays a role in ATP production in the respiratory chain [8].

In the electron transfer pathway, 18 enzyme defects have been reported, and the related disorders and transport proteins are known as fatty acid oxidation disorders. These disorders, with the mechanism of autosomal recessive inheritance, lead to a wide range of symptoms related to the lack of energy in the tissues related to the oxidation of fatty acids, which have sometimes been observed in periods of metabolic crisis [9, 10]. So far, numerous mutations in the *ETFA*, *ETFB*, and *ETFDH* genes have been reported with variable clinical manifestations, which can be classified into three categories: (I) neonatal onset with congenital abnormalities, (II) neonatal onset without congenital anomalies, and (III) late-onset with myopathic phenotype, vomiting, hypoglycemia and rarely metabolic acidosis [11, 12]. Although the genotype-phenotype correlation is significant in GA2 types I and II, it appears poor in type 3. Type I is usually linked to homozygous null mutations, whereas type II is often due to mutations that cause amino acid substitutions, enabling a certain level of remaining enzyme activity [3].

Most neonatal-onset GA2 patients die due to severe postnatal abnormalities such as respiratory failure, cardiomyopathy, hypotension, metabolic acidosis, and profound hypoglycemia [13]. Patients with milder or later-onset disease had more successful treatment than patients with neonatal onset [14], as most GA type I and II patients did not survive due to progressive deterioration [15]. In this study, we conducted a genotypic and phenotypic analysis of a patient with GA2, which resulted in identifying a novel pathogenic variant in the *ETFA* gene. Such studies emphasize the significant role of the whole exome sequencing as a robust technique for diagnosing patients with this heterogenous inherited disorders [16–19].

## Materials and methods

### Ethical consideration and data collection

Ethical approval for data collection was obtained from the Ethics Committee of the Pharmaceutical Sciences Unit of Islamic Azad University, Tehran, Iran (Ethical Approval Code No. IR.IAU.PS.REC.1396.91) and written informed consent was obtained from the legal guardian.

### Patients

**Fig. 1:**
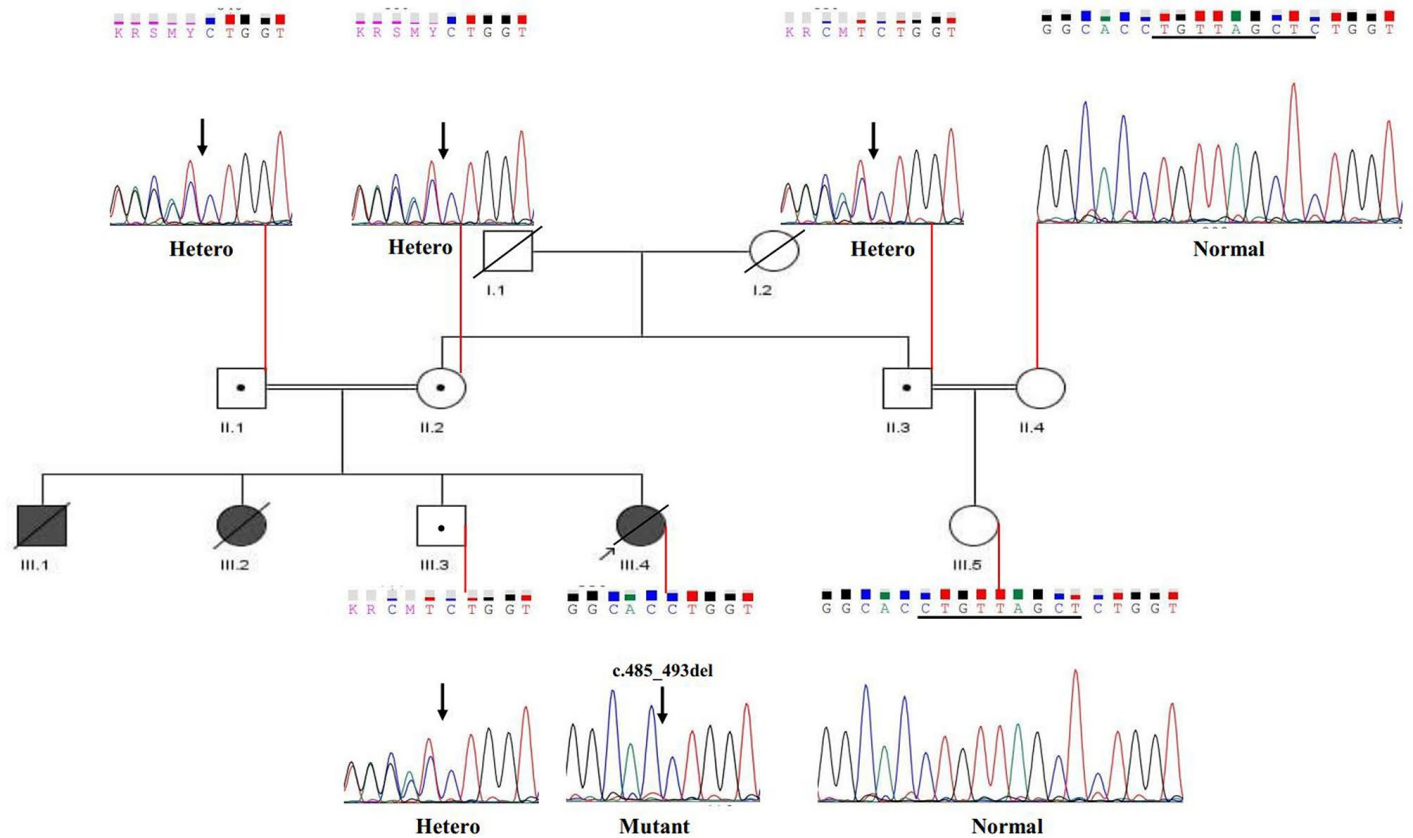
Family pedigree and segregation analysis of c.485_493del: p.E162_T164del variant in the *ETFA* gene. The parents (II.1 and II.2), a healthy son (III.3), and a relative (II.3) are heterozygous carriers of the identified variant in the *ETFA* gene. The fetus (III.4) was reported to be homozygous for this variant. The sequencing illustrates the normal allele in unaffected individuals (II.4 and III.5), as well

Here, we investigated an Iranian family located in Semnan province (central Iran). As shown in Fig. 1, parents (II-1 and II-2) of two deceased neonates (III-1 and III-2) are consanguineous couples who are referred for diagnostic and pre-pregnancy counseling. They had an apparently healthy 17-year-old boy and a history of the death of two neonates, a girl and a boy, 12 and 15 days after birth. Neonates underwent physical and clinical evaluations. Although they appeared physically healthy at birth, they exhibited multiple clinical abnormalities, including liver dysfunction, mild renal abnormalities, elevated urinary organic acid levels, and distinct body odor, and a significant decrease in blood sugar following breast milk consumption. Due to the limitations in gathering data, the biochemical data for the female newborn is detailed in Table 1.

**Table 1:** Biochemical and urinary laboratory tests of 2-day newborn III.2

Biochemistry test	Clinical results	Normal values
AST (SGOT)	127 ↑	Up to 31 U/L
ALT (SGPT)	57 ↑	Up to 31 U/L
Creatinine	1.5 ↑	0.3–0.7 mg/dl
Gamma-GT	212 ↑	Woman < 32 lu/L
CPK	1160 ↑	New born before 5 days: 195–700 U/L
Ammonia	172 ↑	11–51 micromol/l
Blood Glucose	21 ↓	70–105 mg/dl
LDH	2920 ↑	Up to 1100 Iu/L
BUN	5	5–20 mg/dl
Bill.T	8.2 ↑	0.1–1.2 mg/dl
Ca^2+^	6.8 ↓	8.6–10.3 mg/dl
Na^+^	145	135–145 meq/L
K^+^	2.5 ↓	New born: 3.7-5. 9 meq/L
Albumin	3.3 ↓	3.5–5.2 gr/dl
PH	7.2 ↓	7.4
**Urine analysis**	**Clinical results** **(mmol/mol creatinine)**	**Normal values**
Glutaric acid	24,612 ↑	Normal < 19.1 mmol/mol
2-Hydroxyglutaric acid	500 ↑	Normal < 97.3 mmol/mol
3-β-Hydroxybutyric acid	101 ↑	Normal < 9.8 mmol/mol
3-Hydroxyisovaleric acid	146 ↑	Normal < 43.2 mmol/mol
4-Hydroxyphenyllactic acid	19,000↑	Normal < 11.5 mmol/mol
2-Hydroxyisovaleric acid	240↑	Normally not detected
Lactic acid	56,000 ↑	Normal < 564 mmol/mol

AST; Aspartate aminotransaminase, ALT; Alanine aminotransferase, CPK; Creatine phosphokinase, LDH; lactate dehydrogenase, BUN; Blood urea nitrogen, Bill.T; Bilirubin Total

Based on the unusual biochemical results (especially hypoglycaemia and acidosis), the newborns are suspected of having a metabolic disorder, so we proceeded with additional investigations to resolve the matter. In addition, the patient’s ammonia concentration was elevated (172 µmol/L). Based on the biochemical profile, liver function was abnormal, as shown by increased AST (127 U/L) and ALT (57 U/L), although abdominal ultrasonography revealed a normal liver architecture without evidence of structural dysfunction or acute complications.

Additionally, abdominal ultrasonography demonstrated a normal gallbladder, spleen, and pancreas, with no evidence of focal lesions or biliary dilatation. The portal vein diameter was within normal limits. The left kidney measured 48 mm and the right kidney 51 mm, both slightly enlarged and showing increased cortical echogenicity. Mild fullness was observed in the left renal collecting system, with an anteroposterior diameter of the renal pelvis measuring 3.1–8.2 mm. Mild to moderate hydronephrosis was present on the right side, with a renal pelvic diameter of approximately 3.5 mm. The urinary bladder was empty and therefore not evaluable. No free intraperitoneal fluid was detected.

Brain ultrasound revealed no evidence of intraventricular hemorrhage (IVH), intracranial hemorrhage (ICH), or hydrocephalus. The ventricles appeared mildly prominent but within the expected range for a term neonate, and the sulcal and gyral patterns were normal.

Echocardiographic evaluation demonstrated left ventricular dilatation with an ejection fraction of approximately, indicating reduced systolic function. Valvular abnormalities were identified, including mitral regurgitation (MR) and tricuspid regurgitation (TR). In addition, a patent foramen ovale (PFO) was observed, accompanied by bi-ventricular hypertrophy (BVH) (with left ventricular hypertrophy exceeding right ventricular hypertrophy). The atrial situs was solitus with bi-atrial enlargement, and the ventricles showed a d-loop configuration. The great vessels and coronary anatomy were normal. Importantly, echocardiographic assessment revealed mild pulmonary hypertension (PH), consistent with the hemodynamic alterations.

Fundoscopy revealed normal vascularization with intraocular pressure within the normal range. In the right eye, an area of hyperfluorescent changes was observed near the albinoid fovea. Both eyes demonstrated features consistent with retinopathy of prematurity (ROP), Stage II, Zone III.

According to the clinical symptoms of the neonates, their healthy parents were subjected to genetic testing, for which purpose Whole Exome Sequencing (WES) was performed.

### Whole exome sequencing

The karyotype of the parents of two deceased neonates was examined and no chromosomal abnormalities were observed. Then, genomic DNA was extracted from blood samples of seemingly healthy couples following the guidelines of the QIAamp DNA Blood Mini kit (Germany). To explore the genetic etiology, WES was conducted using Illumina Hiseq2000 sequencing technology along with the Agilent SureSelect Human All Exon V7 Kit (Agilent, Santa Clara, USA), targeting all exons of protein-coding genes and several other essential genomic regions. WES was performed for approximately 100 million reads with > 99% sensitivity and > 95% coverage of target regions. After applying various filters, including frequency, intronic regions, 3’-UTR, 5’-UTR, intergenic regions, non-coding segments, and synonymous mutations, the variants were detected.

### Computational analysis of variants and Sanger sequencing

Following the approach taken in the previous study [20], a range of bioinformatics tools, including the BWA aligner, Annova, and GATK, were utilized for enhanced accuracy in reading, functional annotation, and increased throughput of the WES results. In addition, public databases ClinVar, gnomAD, Kaviar and GME were also used. The local population database (BayanGene) was utilized to screen 4,000 unrelated healthy individuals (controls), as well. The potential pathogenic impact of the identified variant was assessed using Mutation Taster and Varsome. Additionally, the Phyre2 tool was employed to examine the influence of the variant on the structural alterations of the ETFA protein in comparison to the wild type protein. ClustalW and NetSurfP-2.0 tools were used to align the ETFA protein sequence among 6 different species and check the accessibility of the residues to the surface and secondary structure of the protein. Given the studied parents’ referral for pre-pregnancy counseling and the background of the death of two children with abnormalities (III.1 and III.2), prenatal diagnosis was advised for the fetus (III.4). To validate the novel variant found, the parents, an apparently healthy child, and a fetus were also analyzed using Sanger sequencing, and the results were examined with the Chromas software. Primers F-5’ AACCATACTCTGCAGAATTC 3’ and R-5’ GATTTCTGGAGTCATACAGC 3’ (PCR product: 621 bp) were designed using the Primer3 program. Additionally, to exclude the chromosomal abnormalities, the chorionic villus sample of fetus was scrutinized to examine the STR markers on chromosomes X, Y, 13, 18, and 21 through the Quantitative Fluorescence-Polymerase Chain Reaction (QF-PCR) approach, and the “3130 Genetic Analyzer” and fragment analysis software were applied.

## Results

WES results revealed a novel heterozygous in-frame deletion variant in exon 6 of the *ETFA* gene (NM_0011127716); chr15:76285661: c.485_493del: p.162_165del in the studied parents (II.1 and II.2 in the Fig. 1). The results obtained from the public databases and the local population database (Bayangen) also did not show any previous reports similar to the variant identified in the *ETFA* gene, which reinforces the novelty of this variant. According to in silico analysis, the novel variant c.485_493del was predicted to be disease causing and likely pathogenesis (Table 2).

**Table 2:** Prediction of pathogenicity of variant identified in *ETFA* gene

Gene	Mutation	Mutation Taster	Varsome	ACMG Criteria Applied
*ETFA* Pathogenic (NM_0011127716)	c.485_493del (E162_T164del)	Disease causing	Likely	PM2, PM4, PP3, PP5

**Fig. 2:**
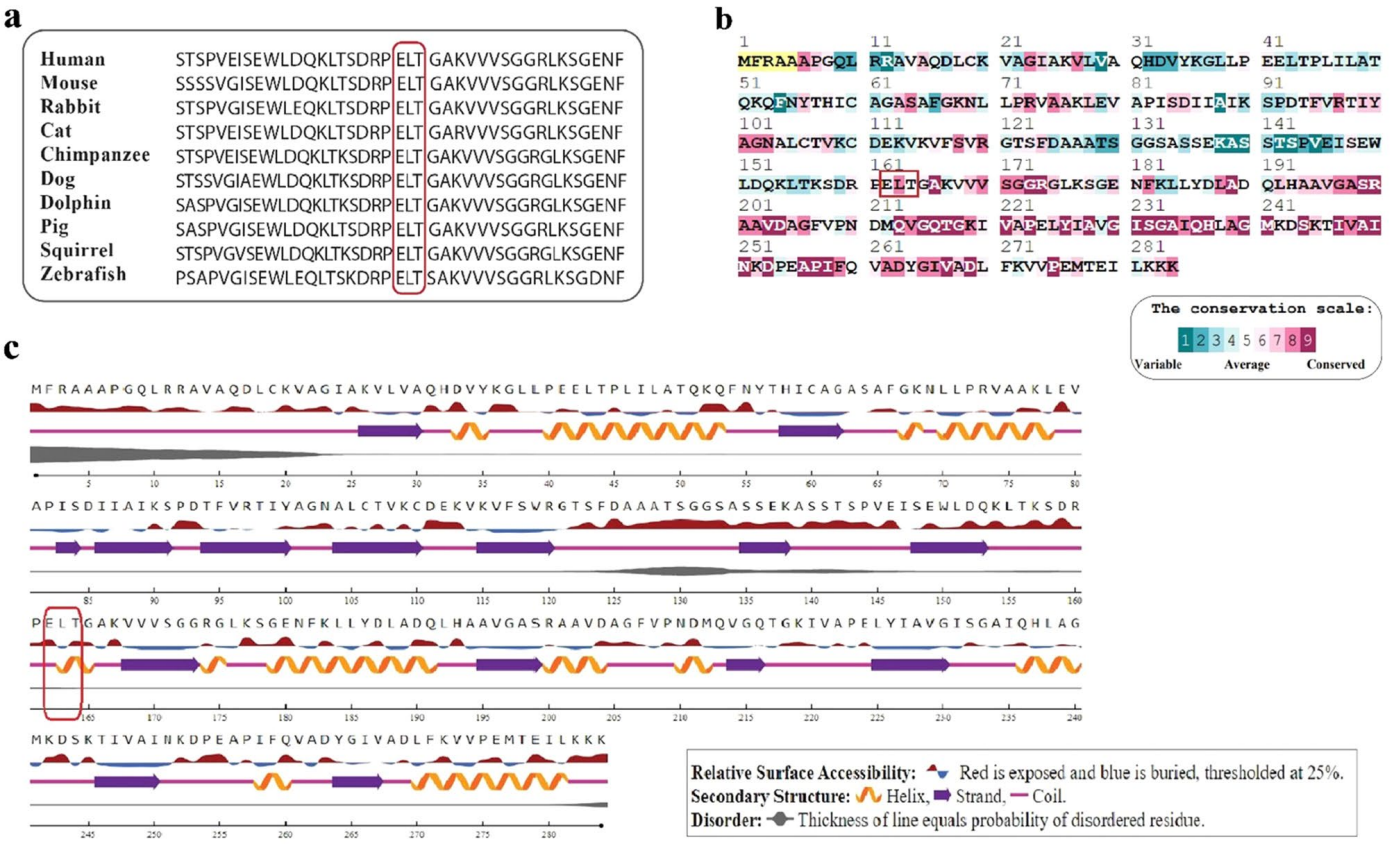
Bioinformatics results of the target sequence in ETFA protein. (**a**) The position of the deleted residues ELT is indicated by a red box. Sequence alignments of ETFA protein in 10 different species indicate high conservation among different organisms. (**b**) According to ConSurf results, glutamic acid, leucine and threonine residues are in conservation scale 6, 8 and 5, respectively. (**c**) The secondary structure of the ETFA protein and the location of glutamine, leucine, and threonine amino acids have been predicted by NetSurfP-2.0

**Fig. 3:**
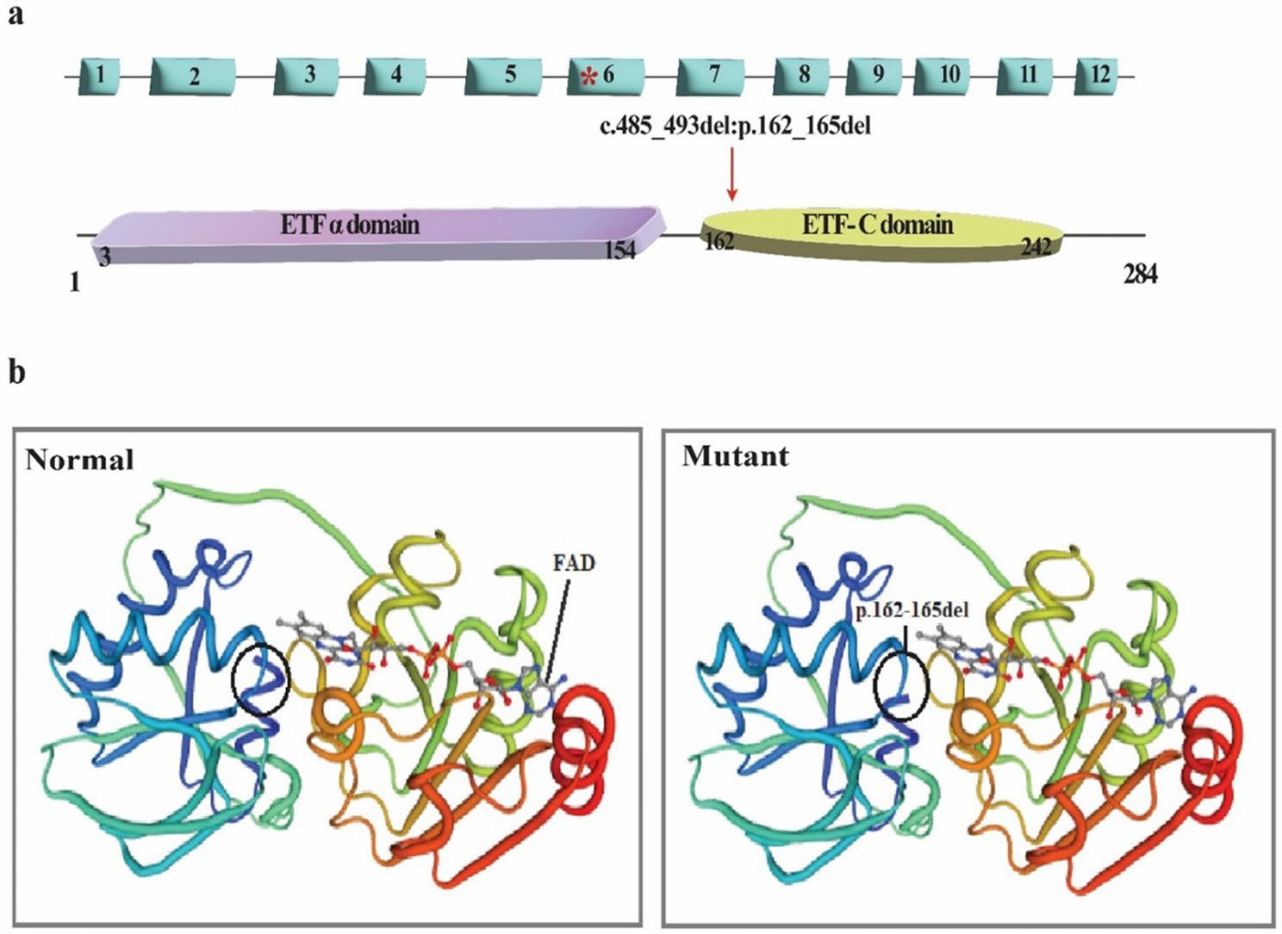
Schematic structure of the ETFA gene/protein and bioinformatic analysis of normal and mutant ETFA protein. (**a**) The position of the studied variant (c.485_493del: p.E162_T164del) is shown at the gene level (top) with an asterisk and in the protein (bottom). (**b**) The effect of deletion variant on ETFA protein structure was predicted by Phyre2 software. The variant resulted in a structural change of the ETF-C domain

As shown in Fig. 1, Sanger sequencing confirmed the presence of the variant in heterozygous form in the *ETFA* gene of the parents studied. The healthy child (III.3) and one relative (II.3) were heterozygous carriers for this variant. These findings are consistent with an autosomal recessive inheritance pattern for the disease. This variant leads to the deletion of 9 nucleotides in the coding region of the gene and, given the importance of this region in protein function, is thought to result in a non-functional protein. In order to further investigate this hypothesis, the amino acid residues present in the deletion region (Fig. 2) and the three-dimensional structure of the protein (Fig. 3) were examined.

As shown in Fig. 2a and b, the position of the ELT residues in the ETFA protein is highly conserved, which most likely indicates the pathogenicity of the variant found. Regarding the positioning of residues, the ET and L are found on the surface and depth of the ETFA protein, respectively. In the secondary structure of the protein, the E residue is in the coil and the LT residues are in the alpha helix (Fig. 2c). Therefore, deletion of this sequence is predicted to lead to a significant change in protein structure and function, as shown in Fig. 2.

The identified variant leads to the deletion of three codons encoding amino acids glutamic acid, leucine, and threonine in the C-terminal domain of the ETFA protein (Fig. 3a). To investigate the effect of the c.485_493del mutation on protein structure, we evaluated the three-dimensional structure of the normal and mutant proteins (Fig. 3b). Modeling the structure of the mutant protein revealed that the deletion of amino acids in the 162–165 region (p.162_165del) leads to changes in the ETF-C functional domain of the protein.

Phenotypes observed in deceased children (according to medical records) correspond to the novel identified variant in *ETFA* gene, and genotype-phenotype correlation studies also confirm it. QF-PCR results showed balanced sets of sex chromosomes and chromosomes 13, 18, and 21 in fetus (III-4). Therefore, fetus is an euploid for the tested chromosomes. The Sanger sequencing confirmed the presence of the variant as heterozygous in the parents (II-1 and II-2), their healthy son (III-3), and a relative (II.3), and as homozygous in the affected fetus (III-4) (Fig. 1). Following the confirmation of the novel variant in the fetus, a legal abortion permit was issued, and the fetus was terminated at the 16th week of its mother’s gestation, with the parents’ consent.

## Discussion

In this study, we identified a novel heterozygous non-frameshift deletion (NM_0011127716: exon 6: c.485_493del: p.E162_T164del) in the *ETFA* gene of the parents of two deceased neonates. Based on the review of the conducted studies, this is the first genetic study of the *ETFA* gene causing the rare disease GA2 in the Iranian population. Our findings showed that this variant leads to the deletion of three codons and consequently the deletion of three amino acids (ELT; glutamic acid, leucine, and threonine) in the C-terminal region of the ETFA protein (Fig. 3b). Since the deleted amino acids (ELT) are located at conserved positions in the ETFA protein (Fig. 2a and b), and the two amino acids L and T are located in the alpha-helical structure of the protein (Fig. 2c), the deletion of this conserved sequence is expected to lead to a serious disruption of the protein structure and function.

These findings are consistent with previous studies, where researchers have shown through complete multiple alignment of ETF family proteins that the C-terminal region exhibits a high degree of sequence identity compared to the N-terminus indicating the critical significance of the C-terminal region in the functionality of ETF family proteins [21]. Moreover, FAD is situated in the space between the two subunits of the ETF protein, with the majority found within the C-terminal region of the α subunit [22]. Consequently, mutations in the C-terminal region can also affect FAD placement and lead to significant impairment of protein function.

GA2 is a genetic disorder with heterogeneous clinical manifestations, ranging from severe neonatal to mild late-onset forms [23, 24]. Clinical manifestations and disorder onset may vary depending on the location and nature of the mutations [25]. According to studies, Neonatal-onset GA2 is usually fatal and presents with severe metabolic decompensations including non-ketotic hypoglycemia, metabolic acidosis, hypotonia, hyperammonemia, coma, and cardiomyopathy. While the duration and age of late-onset forms are highly variable, in adolescents and adults, muscle or cardiac symptoms or episodic vomiting are generally the first manifestations indicative of GA2 [26, 27]. The measurement and analysis of organic acid in the urine show the accumulation of acid in the blood, which is excreted by the kidneys, and in GA2 patients, increased excretion of lactic and glutaric acids along with other hydroxy and dicarboxylic acids has been reported [28]. GA2 treatment mainly consists of a low-protein, low-fat, high-carbohydrate diet, and supplementation with carnitine and riboflavin to stabilize the complex, which has been associated with greater success [15]. To date, 36 distinct pathogenic and likely pathogenic mutations in the *ETFA* gene, associated with a variety of abnormalities, have been reported across different regions (Table 3) [3]. In this study, we present the first identification of a novel mutation in Iran, which can serve as a basis for identifying similar patients in different Iranian populations.

**Table 3:** Clinical characteristics and genotype of patients with pathogenic and likely pathogenic mutations of *ETFA* gene

Origin	Zygosity/ Gender	Clinical phenotype	Age at onset	Nucleotide sequence (aa changes)	Exon/ Intron
Japan	Hetero/ M	Dyspnea, hypoglycemia, hyperammonemia, respiratory distress, metabolic acidosis, vomiting, fatigue without apparent myopathy	0d	c.7 C > T (p.R3X)	E1
Germany	Hetero	Severe hypoglycaemia, Cardiac deterioration	Death: 10 m	c.12_22dup	E1
Portugal	Hetero	Energy depletion and impairment of glucose homeostasis, ketogenesis	NA	c.15_25dup11 (p.Q9Rfs*20)	E1
Belgium	Homo	Neurological deterioration, Metabolic acidosis, Hypoglycemia	Death: D5	c.52 C > T (p.R18X)	E2
Turkey	Hetero	Drowsiness, severe hypoglycaemia, hyperammonemia, metabolic acidosis	6 m	c.73delA (p.I25X)	E2
Turkey	Hetero/ F	Errors of metabolism in asymptomatic newborns, Cystic or dysplastic kidneys	newborn	c.200T > C (p.L67P)	E3
Portugal	Hetero	Biochemical phenotype	NA	c.242 A > C (p.H81P)	E3
Japan	Homo/ M	Reye-like illness, Poor feeding, CPK elevation, Cardiomyopathy	8 m	c.283T > G (p.L95V)	E4
Portugal	Hetero	Biochemical phenotype	NA	c.284T > G (p.L95V)	E4
Portugal	Hetero	Biochemical phenotype	NA	c.346G > A (p.G116R)	E4
Portugal	NA	Biochemical phenotype	NA	c.347G > T (p.G116V)	E4
Limoges, France	Hetero/ M	Facial dysmorphism, Renal cysts associated with severe hypoglycemia, acidosis, hypotonia, hepatomegaly	5y	c.354 C > A (p.N118K)	E5
France	Homo	NA	14y	c.355dupC (p.L119Pfs*10)	E5
Polynesia	Homo	Acute rhabdomyolysis, metabolic acidosis, seizures	3y	c.365G > A (p.R122K)	E5
Belgium	Homo	Neurological deterioration, metabolic acidosis, hypoglycemia	Death: D5	c.431T > C (p.F144S)	E5
Japan	Homo/ F	Liver dysfunction and cardiomyopathy	0d	IVS6-1G > C	I6
Portugal	Hetero	Biochemical phenotype	NA	c.453_470del (p.N152_V157del)	E6
Austria	Homo	Severe hypoglycemia, cardiac arrest	Death: D4	c.470T > G (p.V157G)	E6
Japan	M	Chronic fatigue, recurrent vomiting, metabolic acidosis, weakness	1y	c.478delG (p.D160Mfs*4)	E6
Iranian (This study)	Homo/ F	Severe hypoglycemia, hyperammonemia, metabolic acidosis, hypotonia, drowsiness, hepatic dysfunction, cardiac structural and functional abnormalities, mild renal abnormalities, mild brain abnormalities, ophthalmologic abnormalities	12d	c.485_493del (p.E162_T164del)	E6
France	Hetero	Metabolic coma with hypoglycemia	3 y	c.494T > C (p.V165A)	E6
Portugal	Hetero	Biochemical phenotype	NA	c.502G > T (p.V168F)	E6
Chinese	Hetero/ M	Biochemical phenotype	6 y		
Portugal	Hetero	Biochemical phenotype	NA	c.563-1G > C	E7
France	Homo	NA	14y	c.635T > C (p.L212P)	E7
Limoges, France	Hetero/ M	Severe hypoketotic, hypoglycaemia, hyperammonemia, fatigability, hepatomegaly, energy deficiency	5y	c.652G > A (p.V218M)	E7
Portugal	Hetero	Biochemical phenotype	NA	c.664 + 1del	E7
Portugal	Hetero	Biochemical phenotype	NA	c.667 C > T (p.R223X)	E8
Spain	Homo	Hepatomegaly, bilateral polycystic kidneys, hypotonia, hypertrophic myocardiopathy, seizures metabolic acidosis, hypoglycemia	Death: D2	c.745 C > T (p.R249C)	E9
Portugal	Hetero	Biochemical phenotype	NA	c.751G > A (p.A251T)	E9
Japan	Hetero/ M	Reye-like illness, CPK elevation, cardiomyopathy	1y	764G > T (p.G255V)	E9
Portugal	Hetero	Biochemical phenotype	NA	c.785 A > G (p.Q262R)	E9
France	Hetero	Severe hypoglycemia, cardiac ischemia	18 m	c.797 C > T (p.T266M)	E9
Senegal	Hetero	Severe hypoglycemia	1y		
Germany	Hetero	Severe hypoglycaemia, cardiac ischemia	Death: 10 m		
Turkey	Hetero	Drowsiness, severe hypoglycaemia, hyperammonemia	6 m		
Netherlands	Homo	Coma, hypoketotic hypoglycaemia	3y		
Turkey	Hetero/ F	Myopathic phenotype, chronic fatigue or recurrent vomiting	1d		
Japan	Hetero/ M	Dyspnea, hypoglycemia, hyperammonemia	0d	799G > A (p.G267R)	E9
Portugal	Hetero	Biochemical phenotype	NA	c.809_811del (p.V270del)	E9
Turkey	Hetero/ F	NA	newborn	c.854 A > T (p.Q285L)	E10
France	Hetero	Coma, hypoglycemia, myopathic phenotype, rarely metabolic acidosis	3y	c.875_878del	E10
Portugal	Homo	Psychomotor delay, hypotonia, dystonia	1y	c.963 + 1delG	E11

According to the research and Table 3, the majority of pathogenic variants have been identified in exons 9 and 6 of the *ETFA* gene, suggesting that these exons are prone to mutations [3]. In most cases, ETFA/ETFB/ETF-QO mutations influence the stability of the corresponding messenger RNAs (mRNAs) or the protein network involved in the occurrence of GA2 [29].

Interestingly, hypoglycemic, hyperammonemia, and metabolic acidosis phenotypes have been reported in most patients, and most of them are associated with the neonatal form of GA2. Similarly, in our study, neonates (III.1 and III.2) also showed hypoglycemia, hyperammonemia, metabolic acidosis, drowsiness and hypotonia with strange-smelling secretions without seizures. These findings are consistent with previous reports of patients with neonatal-onset GA2. As previously noted, the majority of GA2 patients with neonatal onset die due to severe abnormalities after birth, in early infancy before GA2 is diagnosed [12]. Considering the irreversible damage of encephalopathic crises and the poor prognosis of acute metabolic crisis in GA1 and GA2 patients, newborn screening is highly recommended for early and correct diagnosis. Current therapeutic mechanisms for GA are based on nutritional therapy [30]. Most GA2 patients have responded well to dietary therapy with L-carnitine, riboflavin or CoQ10.16 supplements [26].

## Conclusion

In summary, we identified for the first time a novel deleterious in-frame variant c.485_493del: p.E162_T164del in the *ETFA* gene of Iranian family. Our study increased the range of *ETFA* gene variants, provided a better understanding of *ETFA* variants on phenotypic outcome and reinforced the clinical importance of this gene in patients with neonatal-onset metabolic disorders. As reported in 2003, there is a distinct connection between the phenotype and genotype in GA2 [5]. Therefore, gathering information on genetic mutations to determine any genotype/phenotype correlations and identifying defective enzymes is necessary for accurate prenatal diagnosis of GA2. Further investigations, particularly assessing the potential functional impact of the novel identified mutation could provide deeper insights into the molecular pathogenesis of this mutation.

## Data Availability

The identified variant of *ETFA* gene in this study is accessible on the ClinVar repository, which can be accessed via the following website: https://www.ncbi.nlm.nih.gov/clinvar/variation/1704301/.

## Abbreviations

ETFA: Electron transport flavoprotein alpha subunit
ETFB: Electron transport flavoprotein beta subunit
ETFDH: Electron transport flavoprotein dehydrogenase
mRNAs: Messenger RNAs
GA2: Glutaric acidemia type II
MADD: Acyl-CoA dehydrogenase deficiency
WES: Whole exome sequencing
